# DUSP1 inhibits cell proliferation, metastasis and invasion and angiogenesis in gallbladder cancer

**DOI:** 10.18632/oncotarget.14815

**Published:** 2017-01-25

**Authors:** Jiliang Shen, Senjun Zhou, Liang Shi, Xiaolong Liu, Hui Lin, Hong Yu, Xiao liang, Jiacheng Tang, Tunan Yu, Xiujun Cai

**Affiliations:** ^1^ Department of General Surgery, Sir Run-Run Shaw Hospital, Zhejiang University, Hangzhou 310016, China

**Keywords:** DUSP1, gallbladder cancer, metastasis, angiogenesis, MMP2

## Abstract

DUSP1/MKP1 is a dual-specific phosphatase that regulates MAPK activity and is known to play a key role in tumor biology. Its function in gallbladder cancer (GBC) remains largely unknown, however. By exploring its activities in two GBC cell lines (SGC996 and GBC-SD), DUSP1 was found to inhibit GBC cell proliferation, migration and invasion. Moreover, DUSP1 inhibited GBC growth and metastasis in nude mice subcutaneously xenografted with SGC996 cells. The tumor suppression appeared to be mediated via the DUSP1-pERK/MAPK-MMP2 signal pathway. Angiogenesis was associated with the tumor metastasis in the mouse model and was impaired by DUSP1, which suppressed VEGF expression. These results suggest that DUSP1 suppresses GBC growth and metastasis by targeting the DUSP1-pERK-MMP2/VEGF axis. Identification of the DUSP1-pERK-MMP2/VEGF signals may provide new biomarkers and/or therapeutic targets to better suppress GBC metastasis in the future.

## INTRODUCTION

Occurring predominantly in elderly women, gallbladder cancer (GBC) is the fifth most common cancer of the digestive tract [1, 2]. Surgery is currently the only recommended treatment. However, GBC presents as an aggressive tumor, and outcomes are poor. Indeed, many of these tumors are unresectable at initial diagnosis, and the 5-year survival rate is less than 10% [3]. A number of studies have already been undertaken in to identify the genes and biological processes critical to GBC initiation and progression [4–10]. A better understanding of the molecular mechanisms underlying gallbladder carcinogenesis and progression could certainly help in the development of novel approaches to the treatment of GBC.

Dual-specificity MAP kinase phosphatase1 (DUSP1/MKP1) is encoded by a highly inducible gene and specifically targets ERK1/2 [11]. An earlier study showed that, in hepatocellular carcinoma, levels of DUSP1 expression correlate inversely with those of phosphorylated ERK, as well as with the proliferation index and microvessel density [12]. Overexpression of DUSP1/MKP1 has been observed in several human epithelial tumors, including prostate, colon and bladder cancers [13–15]. Interestingly, expression of DUSP1/MKP-1 was downregulated in these tumors as the histological grade increased. In addition, DUSP1/MKP1 was found to promote angiogenesis, invasion and metastasis in non-small-cell lung cancer [16]. Opposite effects observed in other tumors suggest the function of DUSP1 in modulating tumor bioactivity is complex and depends on the specific tumor context.

DUSP1 is known to dephosphorylate ERK [11, 17]. Activation of the ERK1/2 pathway promotes cell proliferation [18–22] and leads to malignant transformation [23, 24]. In addition, ERK signaling pathways are over-activated in various human cancers, including cholangiocarcinoma and GBC [25–27]. The function of DUSP1 in GBC has not yet been studied, however. We therefore investigated the potential role of DUSP1 in GBC progression and identified the DUSP1-pERK-MMP2/VEGF signaling pathway to be a key promoter of GBC growth, metastasis and angiogenesis.

## RESULTS

### Expression of DUSP1 is associated with tumor stage and patient survival

**Figure 1:**
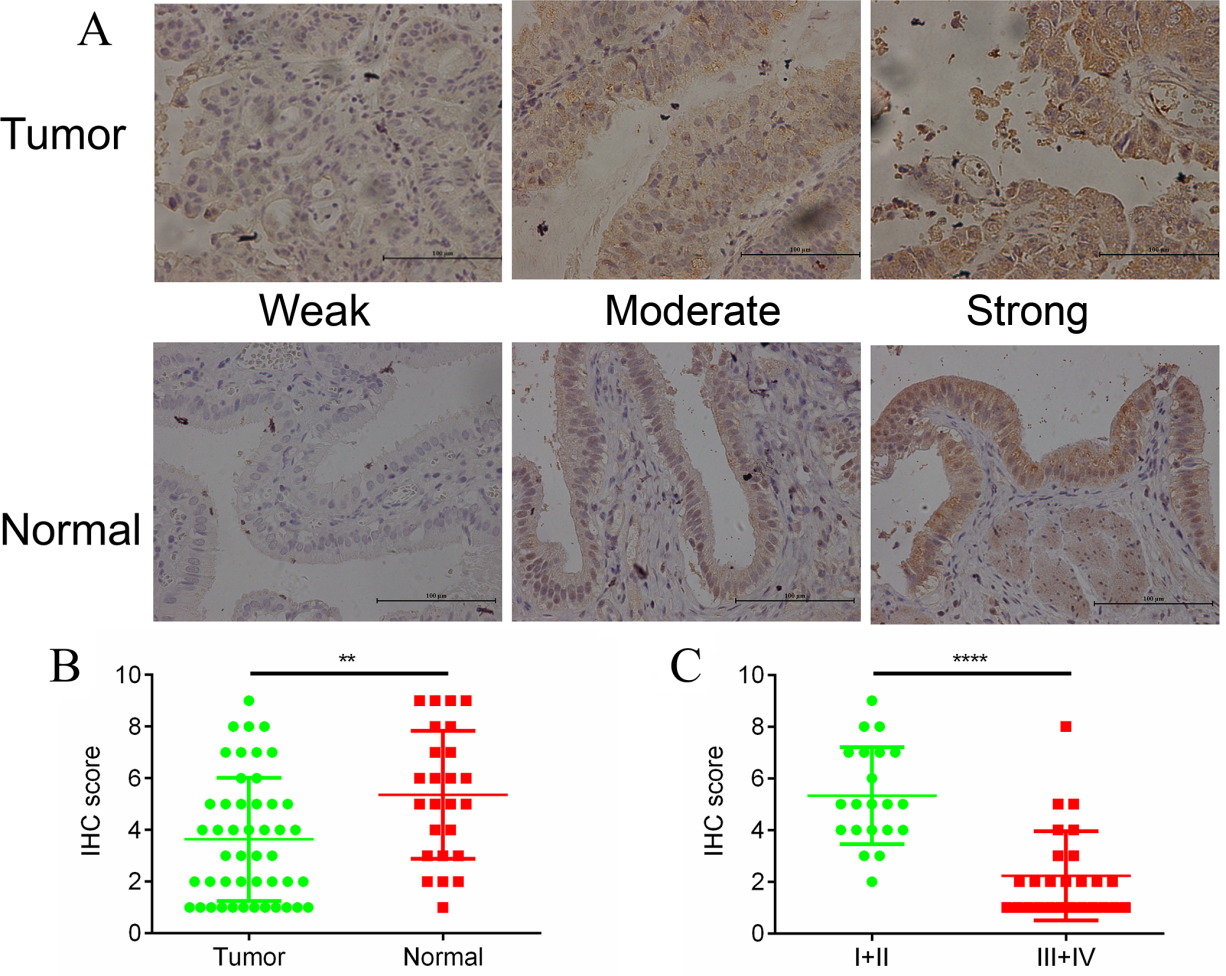
DUSP1 expression in gallbladder tumor/normal tissues and its different expression level in tumors from different tumor stages Positive DUSP1 reactions were mainly localized in the cytoplasm in tumor tissues and normal tissues (**A**). The expressions of DUSP1 were lower in tumor tissues compared with normal tissues (**B**). The expressions of DUSP1 were lower in tumor tissues of late stages (AJCC III and IV) compared with tumor tissues of early stages (AJCC I and II) (**C**). (*P < 0.05, **P < 0.01, ***P < 0.001, ****P < 0.0001).

We first examined expression of DUSP1 in clinical tissue samples obtained from 47 GBC patients. Based on AJCC standards, the tumor was stage I in 6 patients, stage II in 15 patients, stage III in 15 patients and stage IV in 2 patients. Also studied were 25 normal gallbladder tissue samples from gallbladder patients. Interestingly, DUSP1 expression was lower in the tumor tissues than normal tissues (Figure 1A and 1B). In addition, DUSP1 expression was much lower in more malignant tumors (AJCC stages III and IV) than in tissues from less malignant tumors (AJCC stages I and II) (Figure 1C). These results suggest that DUSP1 expression may be correlated with GBC progression.

### DUSP1 inhibits GBC cell proliferation

**Figure 2:**
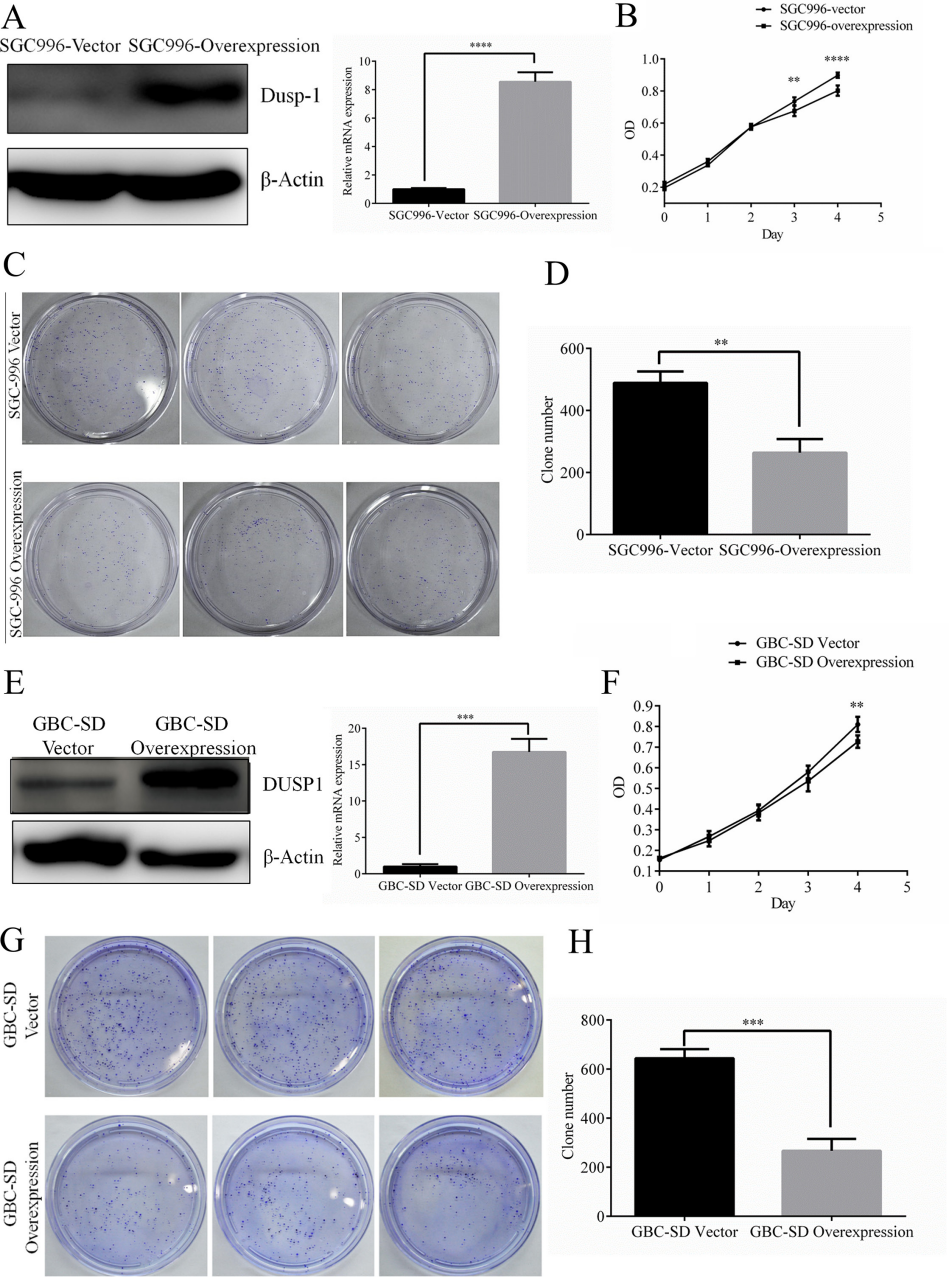
Stable expression of DUSP1 in gallbladder cancer cell lines SGC996 Stable expression of DUSP1 in SGC996 cell (**A**). Proliferation was evaluated by MTT assay (**B**), clone formation assay and relative efficiency of 3 experiments (**C, D**). Stable expression of DUSP1 in gallbladder cancer cell lines GBC-SD. (**E**) Proliferation was evaluated by MTT assay (**F**), clone formation assay and relative efficiency of 3 experiments (**G, H**). (*P < 0.05, **P < 0.01, ***P < 0.001, ****P < 0.0001).

To determine whether DUSP1 directly contributes to GBC cell proliferation, we used a lentiviral overexpression system to generate SGC996 cells stably overexpressing DUSP1 (SGC996-oe cells) (Figure 2A, mRNA level and protein level). MTS assays revealed that the growth rate was significantly decreased in SGC996-oe cells as compared to control cells transduced with empty vector (SBC996-vector cells) on days 3 and 4 after transduction (Figure 2B). Clone formation assays also confirmed the ability of DUSP1 to suppress SGC996 cell proliferation (Figure 2C, 2D). Similar results were obtained with GBC-SD cells (Figure 2D–2H). Together, these results demonstrate that overexpression of DUSP1 suppresses the GBC cell proliferation.

### DUSP1 inhibits GBC cell migration and invasion

**Figure 3:**
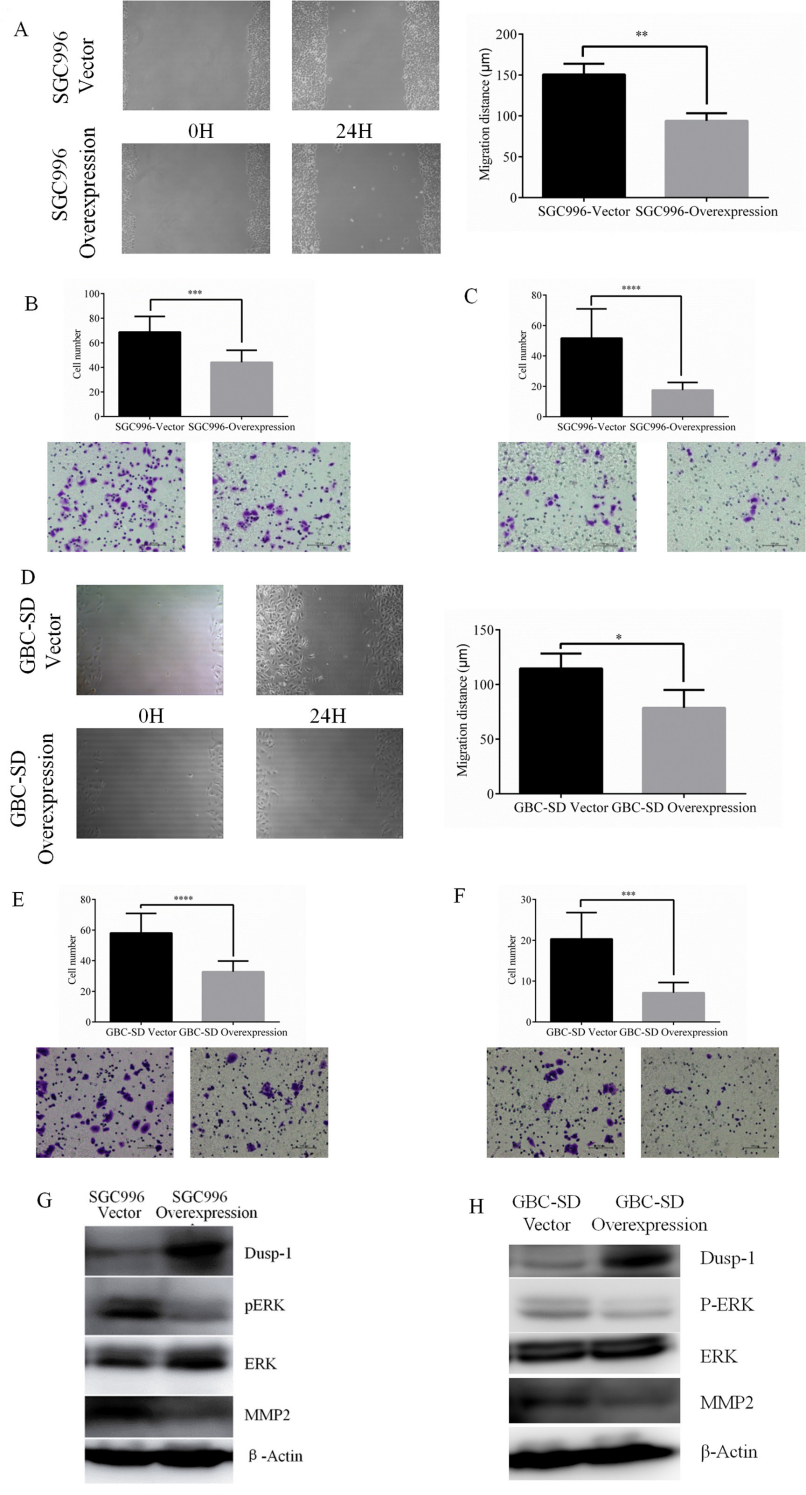
Representative images of wound-healing assay of SGC996 and relative wound space was calculated (**A**) Representative images and quantitatively analysis of migrated SGC996 cells expressing DUSP1 versus vector control (**B**). Representative images and quantitatively analysis of invaded SGC996 cells expressing DUSP1 or vector control (**C**). Representative images of wound-healing assay of GBC-SD and relative wound space was calculated (**D**). Representative images and quantitatively analysis of migrated GBC-SD cells expressing DUSP1 versus vector control (**E**). Representative images and quantitatively analysis of invaded SGC996 cells expressing DUSP1 or vector control (**F**). Western blot analysis results show higher expression of DUSP1 and lower expression of pERK and MMP2 in the two Dusp1 overexpression GBC cells (**G, H**). (*P < 0.05, **P < 0.01, ***P < 0.001, ****P < 0.0001).

In wound-healing assays SGC996-vector cells close the wound (nearly 151 μm) within 24 h, whereas SGC996-oe cells closed a significantly smaller distance (94 μm; *P* < 0.01) over the same time period (Figure 3A). Nonetheless, the growth rates of the two cells lines were similar during the 24 h (Figure 2A and 2C). This indicates the greater wound healing by SGC996-oe cells reflects their greater cell motility, but not growth. This was confirmed by subsequent transwell assays, in which SGC996-vector cells exhibited greater migration and invasion ability than SGC996-oe cells (6812 vs 44 ± 9 migrating cells; 51 ± 19 vs 17 ± 5 invading cells) (Figure 3B and 3C). Again, we obtained similar results using GBC-SD cells (Figure 3D–3F).

### DUSP1 knockdown promotes proliferation, migration and invasion by GBC cells

**Figure 4:**
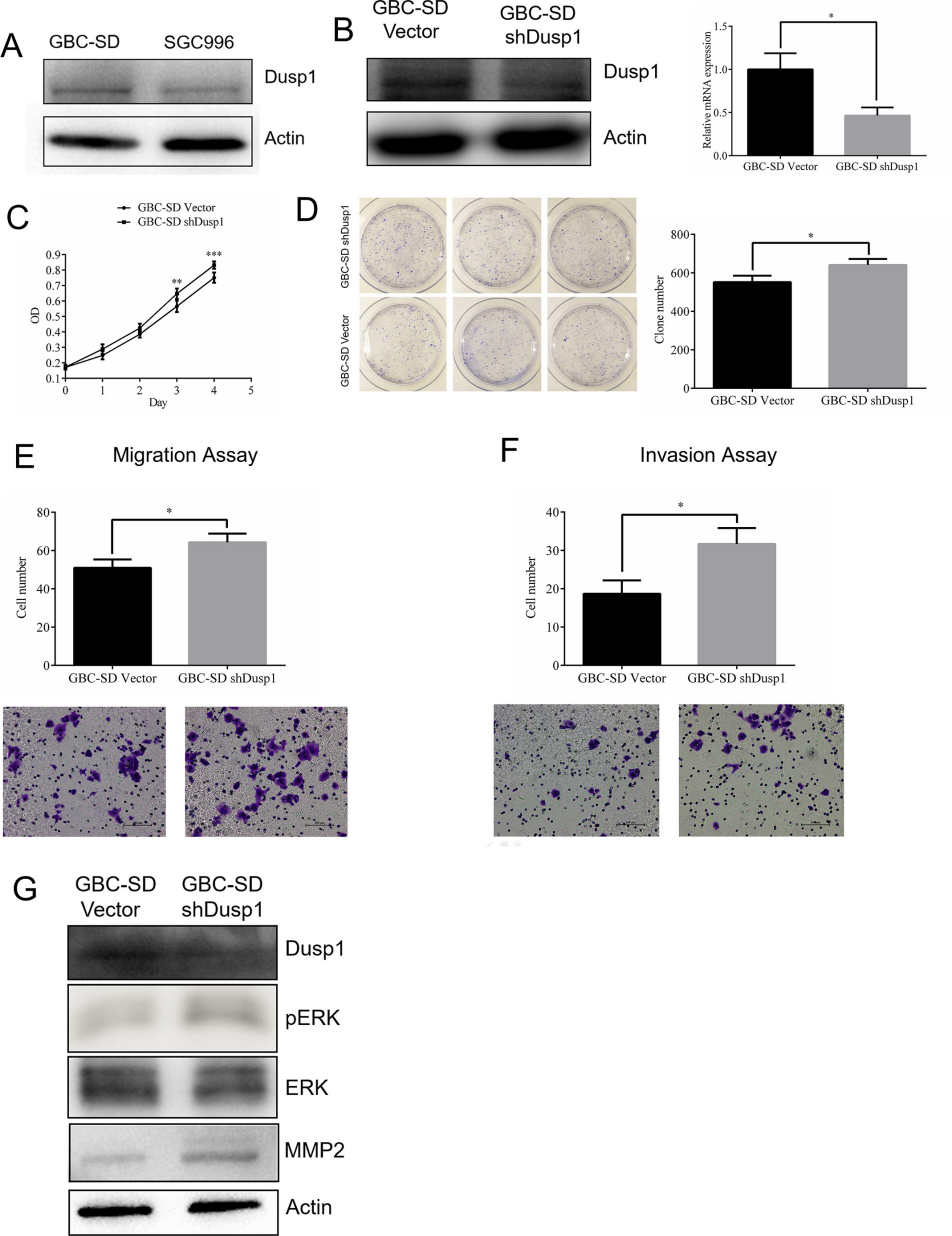
Knocking down DUSP1 in gallbladder cancer cell lines GBC-SD Expression level of DUSP1 was higher in GBC-SD cell (**A**). Knocking down Dusp1 in GBC-SD cell (western blot and Q-PCR) (**B**). Proliferation was evaluated by MTT assay (**C**), clone formation assay and relative efficiency of 3 experiments (**D**). Representative images and quantitatively analysis of migrated GBC-SD cells knocking down DUSP1 versus vector control (**E**). Representative images and quantitatively analysis of invaded GBC-SD cells knocking down DUSP1 or vector control (**F**). Western blot analysis results show lower expression of DUSP1 and higher expression of pERK and MMP2 in the GBC-SD DUSP1 knocking down cells (**G**). (*P < 0.05, **P < 0.01, ***P < 0.001, ****P < 0.0001).

We observed the highest levels of DUSP1 expression in GBC-SD cells (Figure 4A). Knocking down DUSP1 in those cells enhanced both their growth rate and clone formation (Figure 4B–4D). In addition, transwell assays further confirmed that DUSP1 knockdown promotes GBC cell migration and invasion (Figure 4E and 4F).

### Mechanism by which DUSP1 alters GBC cell proliferation, metastasis and invasion

Previous studies [17] indicate that DUSP1 dephosphorylates ERK, and our results revealed that p-ERK levels were consistently reduced in both SGC996-oe and GBC-SD-oe cells (Figures 3G and 3H, 4G). We first hypothesized that DUSP1 might modulate metastasis genes, such as MMP2 and MMP9, by influencing the phosphorylation status of ERK [28, 29]. In some cancers, MMP2 expression is associated with their capacity for metastasis [30–33]. We observed that MMP2 expression is decreased in SGC996-oe and GBC-SD-oe cells, and is increased in DUSP1 knockdown cells. This suggests DUSP1 suppresses GBC cell invasion via a DUSP1-pERK-MMP2 signaling pathway (Figures 3G and 3H; 4G).

### DUSP1 inhibits GBC proliferation in a subcutaneous xenograft mouse model

**Figure 5:**
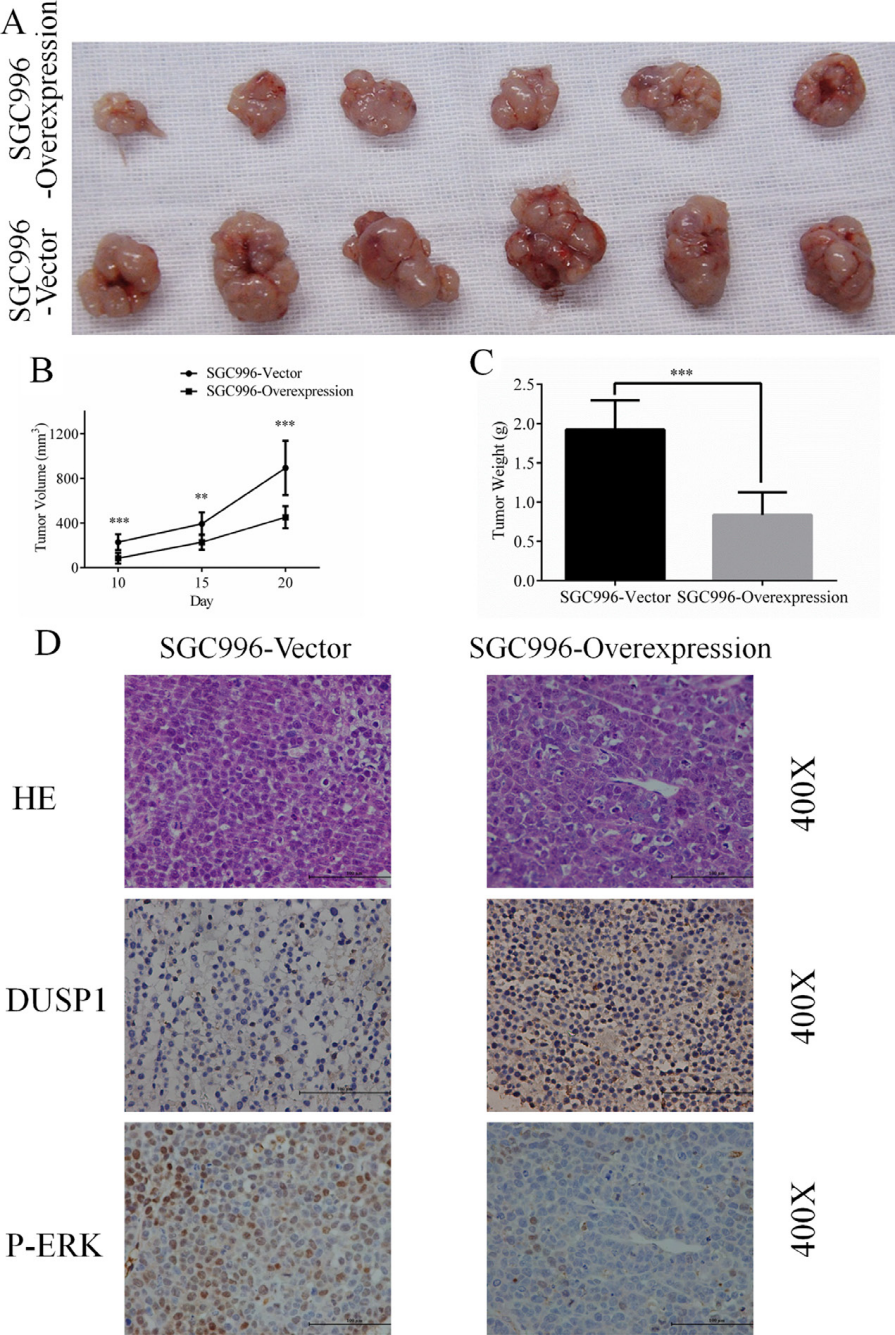
Representative images (**A**), Growth curve (**B**), weight (**C**) of tumors from SGC996-DUSP1 stable cells versus vector control in mice model. HE staining, IHC of DUSP1 and p-ERK were presented below (**D**), original magnification 400×. (*P < 0.05, **P < 0.01, ***P < 0.001, ****P < 0.0001).

To confirm the *in vitro* effects of DUSP1 *in vivo*, we applied a GBC xenograft model by subcutaneously transplanting SGC996-vector or SGC996-oe cells into nude mice randomly divided into SGC996-vector (*n* = 6) and SGC996-oe groups (*n* = 6). As shown in Figure 5A, GBC growth was significantly diminished in mice transplanted with cells overexpressing DUSP1. Both tumor volume and tumor weight were significant smaller in mice receiving SGC996-oe cells (Figure 5B and 5C). Immunohistochemical staining confirmed the enhanced DUSP1 expression in the SGC996-oe cell tumors. On the other hand, stronger p-ERK expression was seen in SGC996-vector cell tumors (Figure 5D).

### DUSP1 inhibits GBC metastasis in the subcutaneous xenograft mouse model

**Figure 6:**
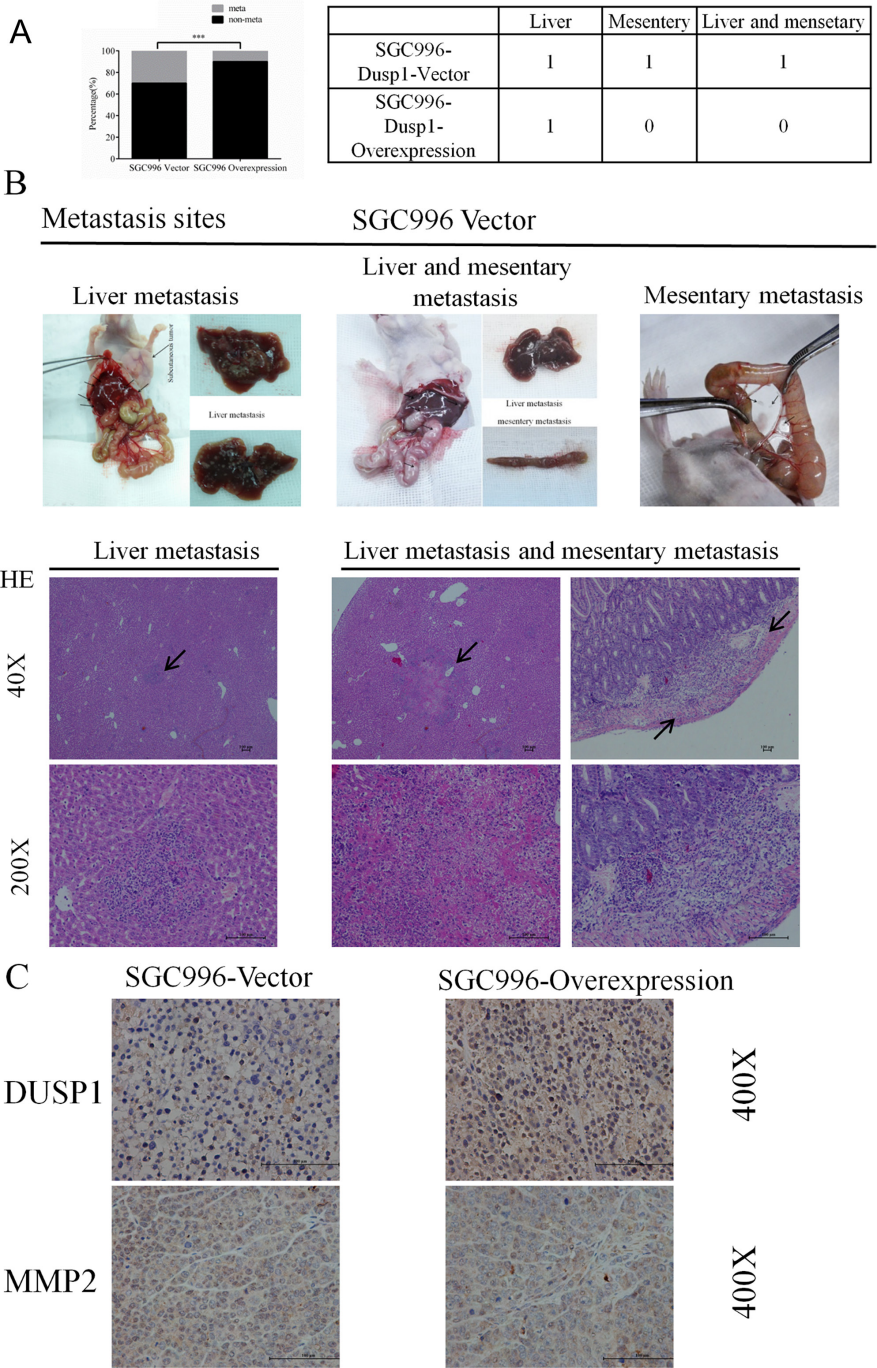
Metastasis incidence (**A**), metastasis sites (**B**) and representive images (**C**) from SGC996-DUSP1 stable cells versus vector control in mice model. HE staining, IHC of DUSP1 and MMP2 were presented below (C), original magnification 40×, 200× and 400×. (*P < 0.05, **P < 0.01, ***P < 0.001, ****P < 0.0001).

To verify the ability of DUSP1 to inhibit metastasis *in vivo*, we applied the same xenograft model, subcutaneously transplanting SGC996-vector (*n* = 10) or SGC996-oe (*n* = 10) cells into nude mice. Six weeks later, we sacrificed the mice and examined them for metastasis. Significantly more mice receiving SGC996-vector cells exhibited metastases than did those receiving SGC996-oe cells (3/10 vs 1/10, *P* < 0.001) (Figure 6A). Metastasis was detected in the liver, mesentery or both in the SGC996-vector group (Figure 6B, marked by arrows). Hematoxylin & eosin staining confirmed the metastatic tumors in mice in the SGC996-oe group originated from the implanted SGC996 cells. Immunohistochemical analysis also revealed stronger MMP2 expression in the SGC996-vector group (Figure 6C).

### DUSP1 modulates angiogenesis in GBC tumors

**Figure 7:**
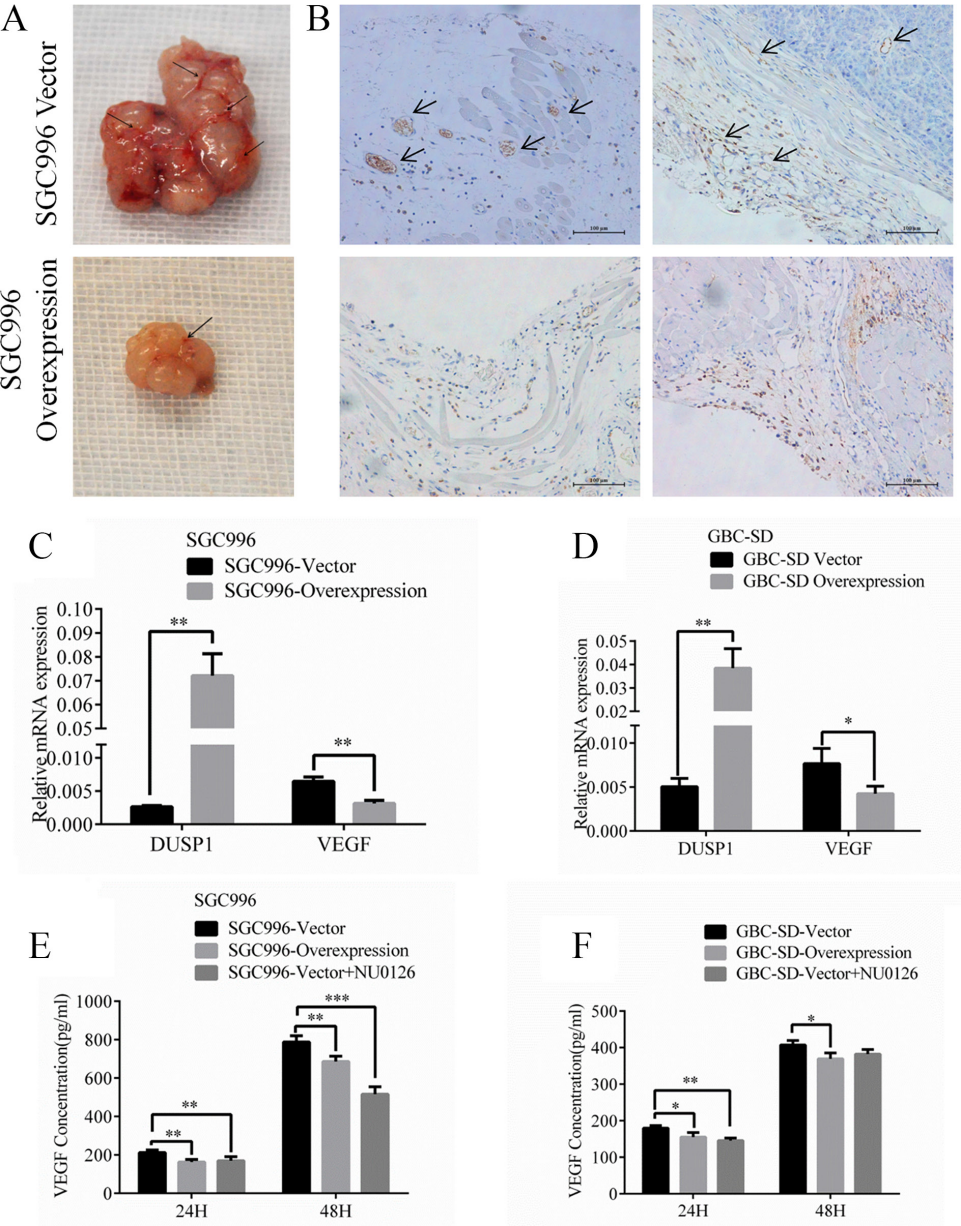
Images of tumor vessels (**A**) from SGC996-DUSP1 stable cells versus vector control in mice model. IHC of CD31 for endothelial cells (**B**) was stained. VEGF relative mRNA (**C, D**) and VEGF concentrations using ELISA detection (E, F) were evaluated representively in SGC996 and GBC-SD stable cells. (*P < 0.05, **P < 0.01, ***P < 0.001, ****P < 0.0001).

During tumor progression, tumor cells acquire the ability to activate angiogenesis [34–36]. Consistent with that effect, more vessels were visible with the naked eye in tumors isolated from mice transplanted with SGC996-vector cells (Figure 7A, marked by arrows). To verify this observation, we identified endothelial cells by staining for CD31 and calculated the microvessel density [37–39], which we found to be higher in SGC996-vector cell tumors than in tumors composed of DUSP1-overexpressing SGC996-oe cells (Figure 7B, marked by arrows). These results demonstrate that DUSP1 expressed in cancer cells significantly suppresses angiogenesis during tumor development. Early studies indicated up-regulation of the Raf/MEK/ERK pathway accelerated Raf/MEK/ERK-mediated VEGF autocrine function [40–42]. When we assessed VEGF expression, we detected lower VEGF levels in SGC996-oe cells than in SGC996-vector cells (Figure 7C). In addition, using an ELISA, we found that less VEGF is secreted from SGC996-oe cells than from SGC996-vector cells (Figure 7D). We obtained similar results with stably transduced GBC-SD cells (Figure 7E and 7F). Based on these findings, we believe DUSP1 inhibits angiogenesis in GBC tumors by reducing secretion of VEGF.

## DISCUSSION

DUSP1 is reportedly involved in the progression of some tumors, including prostate, colon and liver cancers [11, 13, 14]. DUSP1 was found to promote carcinogenesis in prostate and pancreatic cancers, but inhibit carcinogenesis in hepatocellular carcinoma. In prostate, ovarian, colon and gastric cancers, progressive loss/reduction of DUSP1 is detected with increasing histological grade, which indicates DUSP1 may act a tumor suppressor in those settings. The function of DUSP1 during GBC progression and metastasis remains largely unknown. In this study, we first demonstrated that DUSP1 expression contributes to GBC cell proliferation, migration and invasion. Immunohistochemical staining for DUSP1 in 47 tumor samples from GBC patients and 25 samples of normal gallbladder tissue indicated lower expression levels in tumor tissues. Moreover, when we divided the tumor samples into two groups according to tumor stage (Group I (AJCC stage I and II) and Group II (AJCC stage III and IV)), we found that DUSP1 levels were lower in Group II. This further suggests DUSP1 acts to suppress GBC progression. Consistent with that idea, we found that overexpressing DUSP1 inhibited GBC cell proliferation, migration and invasion while DUSP1 knockdown had the opposite effect both *in vitro* and *in vivo*. In addition, DUSP1 also suppressed angiogenesis in GBC tumor.

Examination of the underlying mechanism of DUSP1 action suggests it dephosphorylates ERK/MAPK in GBC cells, thereby influencing expression of genes downstream of ERK/MAPK. Activation of ERK/MAPK promotes cell proliferation and metastasis in multiple cancers [20, 22–25]. Our results indicate DUSP1 may function through ERK/MAPK to alter expression of MMP2 and VEGF and thus influence GBC growth, metastasis and angiogenesis.

In sum, we demonstrated that DUSP1 suppresses GBC cell proliferation and metastasis as well as angiogenesis. These effects are likely linked to downregulation of MMP2 and VEGF related to the reduction p-ERK levels. These findings may provide valuable information for future development of new approaches to the treatment of GBC.

## MATERIALS AND METHODS

### Tissue samples

Gallbladder cancer and normal gallbladder clinical samples were obtained from Department of General Surgery, Sir Run-Run Shaw Hospital, Zhejiang University, Hangzhou, China, with signed Informed Consent for the use of their tissues for scientific research. Clinical samples were from 47 GBC patients with different tumor stages (Ι stage: 6 patients; II stage: 15 patients; III stage: 15 patients; IV stage: 2 patients) according to AJCC standard and 25 normal gallbladder tissues that from gallbladder patients. The current study was approved by the Institutional Review Board. Survival information of the patients was obtained through letters and phone calls.

### Cell culture

Human gallbladder cancer cell lines GBC-SD were obtained from the Type Culture Collection of the Chinese Academy of Sciences (Shanghai, China). SGC996 was provided by Dr. Ying-Bin Liu's lab at Xin Hua Hospital Affiliated to Shanghai Jiao Tong University School of Medicine, China. GBC-SD and SGC996 were cultured in RPMI-1640 containing penicillin and streptomycin, supplemented with 10% fetal bovine serum (FBS). All cell lines were cultured in a 5% (v/v) CO2 humidified incubator at 37°C.

### Construct stable expression gallbladder cancer cell lines

The DUSP1 expression plasmid was generated by cloning DUSP1 cDNA, into retroviral transfer plasmid pWPI to generate plasmid pWPI-DUSP1, DUSP1 knocking down plasmid was from GeneCopoeia (#:HSH004498-CH1). To generate DUSP1 overexpressing and knocking down cells, GBC-SD and SGC996 cells were transfected with lentiviral vectors, pWPI-DUSP1/pWPI-Vec or shDUSP1/Vector, the psAX2 packaging plasmid, and pMD2G envelope plasmid were transfected into 293T cells using the standard calcium phosphate transfection method for 48 hr to get the lentivirus soup. Collected the lentivirus soup and frozen in –80°C for use. The cells were transfected using Lipofectamine 2000 (Invitrogen). Lentiviral supernants were then collected to infect gallbladder cancer cells. After viral infection, the media was replaced with normal culture media. The stable cells were selected and confirmed by quantative real-time PCR (qPCR) and western blot.

### Quantitative real-time PCR

For RNA extraction, total RNAs were isolated using Trizol reagent (Invitrogen, Grand Island, NY). 1–2 μg of total RNA was subjected to reverse transcription using Superscript III transcriptase (Invitrogen, Grand Island, NY). Quantitative real-time PCR (qRT-PCR) was conducted using a Bio-Rad CFX96 system with SYBR green to determine the mRNA expression level of a gene of interest. Expression levels were normalized to the expression of GAPDH mRNA. miRNAs were isolated by using PureLink^®^miRNA kit. In brief, 50 ng small RNA was process for poly A addition by adding 1 unit of polymerase with 1 mM ATP in 1 × RT buffer at 37°C for 10 minutes in 10 ìl volume, and then heat inactivate at 95°C for 2 minutes, add 50 pmol anchor primer to 12.5ìl, incubate at 65°C for 5 minutes, last step cDNA synthesis, add 2ìl 5× RT buffer, 2ìl 10 mM dNTP, 1ìl reverse transcriptase to total 20ìl, incubate at 42°C for 1 hour 25. The sequences of GAPDH primers are: forward 5′-GGAGTCAACGGATTTGGT-3′, reverse: 5′-GTGATGGGATTTCCATTGAT-3′. The sequences of MMP2 are: forward 5′-CAAGAACAAGAAGACA TACATC-3′, reverse: 5′-CTCCAACTTCAGGTAATA CG-3′. The sequences of DUSP1 are: forward 5′-CCT GAC AGC GCG GAA TCT -3′, reverse: 5′-GAT TTC CAC CGG GCC AC -3′.

### Western blot analysis

Cells were lysed in RIPA buffer and proteins (20–50 μg) were separated on 10% SDS/PAGE gel and then transferred onto PVDF membranes (Millipore, Billerica, MA). Membranes were blocked with 5% BSA and incubated with appropriate dilutions of specific primary antibodies against ACTIN (Santa Cruz, # sc-130301),DUSP1 (Abcam,# ab1351), MMP-2 (abcam, #ab86607), ERK (CST #4695), p-ERK (CST #4376). The blots were incubated with HRP-conjugated secondary antibodies and visualized using ECL system (Thermo Fisher Scientific, Rochester, NY).

### MTS assay

Stable transfected cells (4 × 103) were seeded on a 96-well plate with 3 replicate wells and allowed to incubate for 96 hr. After incubation, cell viability was assessed Every 24 hr utilizing the tetrazolium-based MTS colorimetric assay (CellTiter 96 cell proliferation assay kit; Promega, Madison, WI, USA) according to the manufacturer's instructions. All experiments were performed at least in triplicate on three separate occasions. A dose-response curve was plotted.

### Clone formation assay

In clone formation assay, cells were plated in 10 cm plates at a density of 1 × 103 cells/plate. Cells were maintained in RPMI-1640 containing penicillin and streptomycin, supplemented with 10% fetal bovine serum (FBS). All cell lines were cultured in a 5% (v/v) CO2 humidified incubator at 37°C for two weeks.

### Migration assay and invasion assay

Cell migration assays were performed according to the manufacturer's protocols. Cells were trypsinized and resuspended in serum-free media and subsequently seeded in transwell chambers (BD Falcon, USA). Then, the cells were cultured for 24 h followed by PBS washes, fixation with 4% formaldehyde (Sigma), and 0.1% crystal violet staining. The unmigrated cells were removed using cotton swabs, and the migrated cells were counted.The invasion capability of gallbladder cancer cells was determined by the transwell assay. Before seeding the cells, 10 mL of Matrigel (BD, Inc) was dissolved in 50 mL serum-free DMEM or RPMI-1640 medium, applied to upper chamber of 8 mm-pore-size polycarbonate membrane filters (Corning, Inc., Corning, NY), and put into the incubator for 5 hours. Gallbladder cancer cells were then harvested and seeded with serum-free RPMI-1640 medium into the upper chamber at 1×105 cells/well, and the bottom chamber of the apparatus contained RPMI-1640 medium with 10% FBS, and then transwells incubated for 48 h at 37°C. Following incubation, the invaded cells attached to the lower surface of the membrane were fixed by 4% paraformaldehyde and stained with 1% toluidine blue. Cell numbers were counted in six randomly chosen microscopic fields (100×) per membrane. *P* values were calculated using an unpaired two tailedt test.

### ELISA

ELISA kits were used to measure the concentrations of VEGFA (R&DSystems) according to the manufacturer's instructions.

### *In vivo* growth and metastasis studies

Female 6–8 weeks old athymic nude mice were purchased from NCI. 20 mice were divided into 2 groups (*n* = 20). 1 × 10^6^ GBC996 stable cells (mixture with Matrigel, 1:1) were injected subcutaneusly. The first group mice were injected with vector cells; The second group were injected with over-expression DUSP1 ones; The tumor volume (V) was calculated according to the formula: V= (W^2^×L)/2. The mice were anaesthetized after experiment, and tumor tissue was excised from the mice and weighted. Another 20 mice were divided into 2 groups (*n* = 20). 1 × 10^6^ GBC996 stable cells (mixture with Matrigel, 1:1) were injected subcutaneusly. The first group mice were injected with vector cells; The second group were injected with over-expression DUSP1 ones; after one month, mice were sacrificed and tumor metastases to distant organs were analyzed. All animal experiments were performed humanely in compliance with guidelines reviewed by the Animal Ethics Committee of the Biological Resource Centre of the Agency for Science, Technology and Research. The metastasis in liver was further examined by H&E staining.

### H&E and immunohistochemical (IHC) staining

Tissues were fixed in 10% (v/v) formaldehyde in PBS, embedded in paraffin, and cut into 4ìm sections and used for H&E staining and IHC staining with human antibodies (concentrations of antibodies: DUSP1 1:150; MMP2 1:150; pERK 1:150). To enhance antigen exposure, the slides were treated with 1×EDTA at 98°C for 10 min for antigen retrieval. The slides were incubated with endogenous peroxidase blocking solution to inhibit endogenous peroxidase, and then were incubated with the primary antibody at room temperature for 60 min. After rinsing with Tris-buffered saline, the slides were incubated for 45 min with biotin-conjugated secondary antibody, washed, and then incubated with enzyme conjugate horseradish peroxidase (HRP)-streptavidin. Freshly prepared DAB (Zymed, South San Francisco, CA) was used as substrate to detect HRP. Finally, slides were counter stained with hematoxylin and mounted with aqueous mounting media.

### Statistical analysis

Data are expressed as mean ± SEM from at least 3 independent experiments. Statistical analyses involved paired *t*-test with SPSS 17.0 (SPSS Inc., Chicago, IL). *In vivo* study, measurements of tumor metastasis among the three groups were analyzed through one-way ANOVA coupled with the Newman-Keuls test. *P* < 0.05 was considered statistically significant.

36 Harlozinska A. Progress in molecular mechanisms of tumor metastasis and angiogenesis. Anticancer Res. 2005; 25:3327–3333.
